# New Chironomidae (Diptera) with elongate proboscises from the Late Jurassic of Mongolia

**DOI:** 10.3897/zookeys.130.1555

**Published:** 2011-09-24

**Authors:** Elena D. Lukashevich, Andrey A. Przhiboro

**Affiliations:** 1 Borissiak Paleontological Institute, Russian Academy of Sciences, Moscow, Russia; 2 Zoological Institute, Russian Academy of Sciences, St.Petersburg, Russia

**Keywords:** Diptera, Chironomidae, fossil, proboscis, feeding, new species, Mongolia, Late Jurassic

## Abstract

Four new species of Chironomidae with well-developed elongate proboscises are described from a Late Jurassic site Shar Teg in SW Mongolia. These are named *Cretaenne rasnicyni* **sp. n**., *Podonomius blepharis* **sp. n.**, *Podonomius macromastix* **sp. n**., ?*Podonomius robustus* **sp. n.**

## Introduction

The present paper continues a series of articles with descriptions of Diptera from the Late Jurassic Shar Teg site (e.g. Kalugina 1992, Lukashevich 2009). The Upper Jurassic lacustrine deposits of Shar Teg Beds outcrop at Ulan Malgait Mt., 4–5 km west of Shar Teg Mt., 100 km ESE of Altai Somon, Gobi-Altai Aimag, SW Mongolia. The fossil assemblage of Shar Teg includes a diverse and abundant complex of flora and fauna (Gubin and Sinitza 1996).

About 600 identifiable dipteran fossils are known among 3000 fossil insects collected at Shar Teg. Up to now, members of two culicomorph families are described from this locality, Dixidae (Lukashevich 1996) and Chaoboridae (Lukashevich in press). The representatives of Culicidae and Ceratopogonidae (unknown in Jurassic beds), Simuliidae (rare Jurassic finds) and Thaumaleidae (one fossil from Transbaikalia, J3–K1) are not found in Shar Teg.

The Mesozoic records of Chironomidae are numerous, and usually it is the aquatic immatures that are dominant (Kalugina and Kovalev 1985, Jell and Duncan 1986, Kalugina 1993). In Shar Teg, the Chironomidae is one of the most numerically abundant groups: about fifty impressions of adults and twenty pupae and empty pupal exuviae have been collected (undoubted larvae are absent), but due to poor or fragmentary preservation most adults have not been determined even to subfamily. Therefore only several specimens are described herein (pupae will be described later). This chironomid assemblage “very much resembles the stranded corpses of adults and pupae left beside a falling stream, or on the wave swept shore of a lake” (P.S. Cranston, pers. comm.).

The adults of nearly all extant chironomid midges have reduced mouthparts and so their common name is “non-biting midges”. However the presence of toothed mandibles in a chironomid midge was recognized first by Downes and Colless (1967), and now they are described in two recent genera of Podonominae, *Archaeochlus* Brundin, 1966 and *Austrochlus* Cranston, 2002 known only from Australia and southern Africa (Cranston et al. 1987, Cranston et al. 2002). Their mandibles closely resemble those of many insectivorous predatory Ceratopogonidae such as *Probezzia* Kieffer, 1906; however, until females are observed feeding, the question will remain unresolved. A culicomorphan of uncertain affinity with a long proboscis is described from the Upper Triassic Cow Branch Formation (Late Carnian) of Virginia, USA (Blagoderov et al. 2007). Recently, functional mandibulate mouthparts are reported in females (and sometimes even in males) of several extinct genera of Chironomidae from Early Cretaceous Lebanese amber (Azar et al. 2008).

New chironomids with biting mouthparts from Shar Teg are described herein. These fossils are housed in the Borissiak Paleontological Institute, Russian Academy of Sciences, Moscow (PIN). Photographs were made using a Leica MZ 9.5 stereomicroscope with a Leica DFC420 digital camera, with further correction using Adobe Photoshop® CS 9.0 software. Measurements were made with an ocular micrometer in a Leica stereomicroscope. Morphological terminology and measurements mainly follow Sæther (1980). Vein nomenclature is after Wootton and Ennos (1989), followed by Shcherbakov et al. (1995): the chironomid veins traditionally named MCu and An are in fact bM3+4 (*tb* of Kalugina) and CuP, respectively. For further details regarding the mentioned fossil localities, see Rasnitsyn and Quicke (2002).

## Systematics

### Family Chironomidae Newman, 1834. Subfamily ?Aenneinae Ansorge, 1999

#### Genus Cretaenne

Azar, Veltz & Nel, 2008

http://species-id.net/wiki/Cretaenne

Cretaenne Azar et al. 2008: 688. Type species *Cretaenne kobeyssii*Azar et al. 2008: 689.

##### Notes.

The genus was established based on two species fromEarly Cretaceous Lebanese amber. The specimens under description are assigned to this genus due to functional blade-like laciniae and mandibles in females, postnotum with a longitudinal groove, reduced hind tibial comb, and peculiarities of wing venation (vein C long and reaching wing tip; Sc not terminating in wing margin; R2 present; cell between divergent R2+3 and R4+5 very broad; R4+5 almost straight; bM3+4 present; *r-m*,bM3+4 and *m-cu* aligned; *m-cu* connecting CuA proximal to *r-m*). In the new species, tibial spurs are probably present; however, their structure remains unclear due to the state of preservation. The structure of claws is important for the determination of Lebanese species, but claws are not visible in the Mongolian specimens as well as the details of chaetotaxy (e.g., on pedicel) and therefore not mentioned in the description.

#### Cretaenne rasnicyni

Azar, Veltz & Nel, 2008 sp. n.

urn:lsid:zoobank.org:act:1F783221-19EE-43C3-8764-45B6B34E0CB4

http://species-id.net/wiki/Cretaenne_rasnicyni

##### Etymology.

Named in honour of an outstanding Russian palaeoentomologist Dr A.P. Rasnitsyn.

##### Material examined.

**Holotype:** part and counterpart of well-preserved female PIN 4270/2379±, SW Mongolia, Shar Teg (443/1); Late Jurassic. **Paratypes:** impressions of two females PIN 4270/2367±, 2459, from the same outcrop.

##### Diagnosis.

The new species is distinguished from both known species of *Cretaenne* by the longer proboscis (more than half of the head height and about twice the clypeus height) and the longer Rs stem.

##### Description.

Female. *Measurements* (mm): Total length 2.1–2.7 (holotype 2.3); thorax length ca. 0.8, width ca. 0.4; wing length 2.1–2.3 (holotype 2.1); abdomen length 1.35–1.8 (holotype 1.5). Total length / wing length 1.1.

##### Holotype

**Figure 1.**
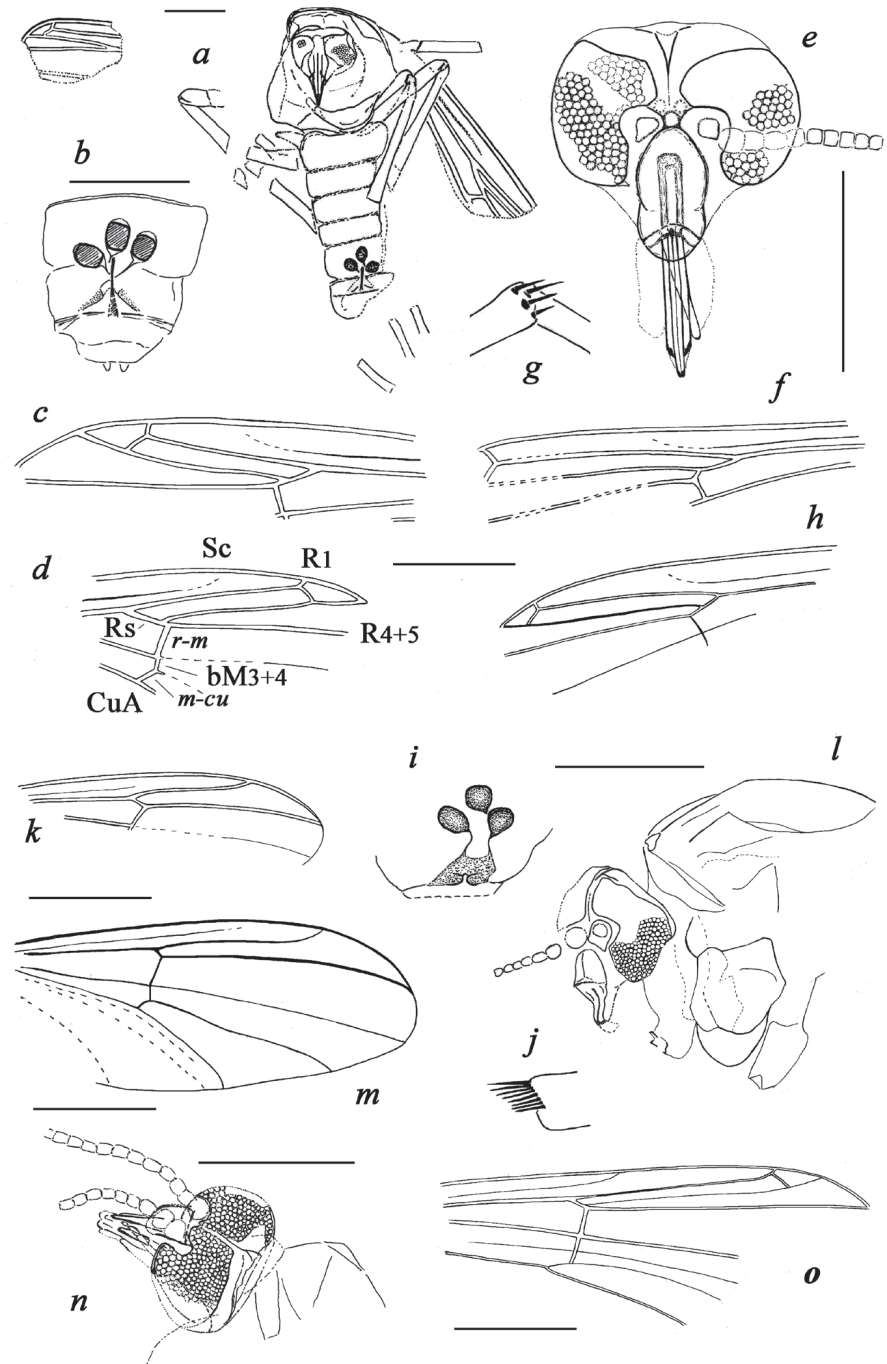
**a–h** *Cretaenne rasnicyni* sp. n. **a–d** holotype (**a** total habitus **b** abdominal apex **c, d** wings) **e–f **paratype PIN 4270/2367 (head and wing) **g–h** paratype PIN 4270/2459 (apex of midtibia and wing) **i–l** *Podonomius blepharis* sp. n., holotype (**i** abdominal apex **j** hind tibial apex **k** wing **l** head and thorax) **m–n ***Podonomius macromastix* sp. n., holotype (wing and head) **o** Tanypodinae inc. sed., PIN 4270/2324; all from Shar Teg, J3. Scale bar 0.5 mm except for **g**, **j** without scale.

**Figure 2.**
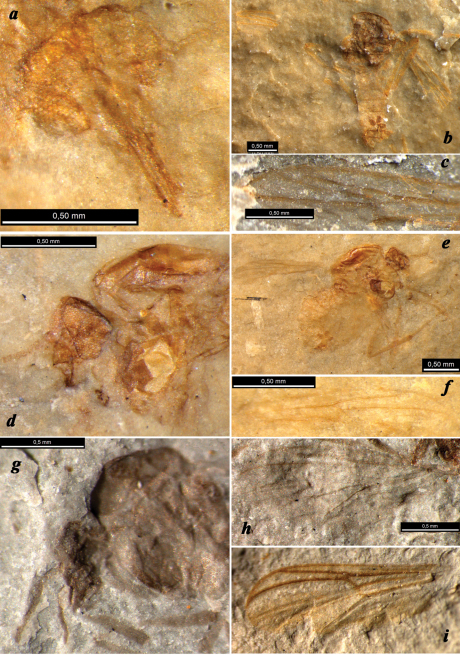
Jurassic Chironomidae: **a–c** *Cretaenne rasnicyni* sp. n. (**a** paratype PIN 4270/2367, female head under alcohol **b–c** holotype **b** female habitus, positive impression under alcohol **c** wing, negative impression) **d–f** *Podonomius blepharis* sp. n., holotype (**d** female head and thorax, negative impression under alcohol **e** female habitus, positive impression under alcohol **f** wing, negative impression under alcohol) **g–h** *Podonomius macromastix* sp. n., holotype (female head and wing); all from Shar Teg, J3 **i** holotype of ?*Podonomius rotundatus* Kalugina, 1985, Kubekovo, J2.

(Figs 1a–d, 2b–c). *Coloration*. Thorax dark, abdomen and legs lighter, at least some legs with slightly darker apices of femora and tibiae. *Head* ca. 550 μm wide, ca. 400 μm high to lower eye margin. Scape ca. 100 μm, pedicel ca. 30 μm in diameter. Clypeus ca. 200 μm wide, ca. 250 μm high. Proboscis with visible part ca. 350 μm long (possibly, without apex), tapering, sclerotized. *Thorax*. Postnotum wider than long, ca. 200 μm long, possibly with longitudinal median groove. *Wing* longer than abdomen, Sc clearly up to Rs level, thinning distally, possibly not reaching C (apical part of Sc not discernible). Vein C and radial veins strong, coloured (R2+3 thinner then others), as well as proximal sections of M and CuA, *r-m* and bM3+4. Long stem Rs subequal to *r-m*; R4+5 8–9 times as long as Rs; R2 distinctly longer than dR1. VR 1.1. *Legs* (lengths not measurable). Femora (mid- and hind) widened to apex, maximum 120 μm wide, with thin sclerotized ridge ventrally near apex. Tibiae (mid- and hind) apically 80–100 μm wide. Tarsi not preserved. *Abdomen*. Abdominal segments II–V: ca. 200 μm long, 500–600 μm wide. Three large subequal oval sclerotized spermathecae 140–150 μm long, ca. 100 μm wide, with necks (probably, short). Gonapophysis IX distinctly visible, sclerotized, with notum ca. 200 μm long, 15 μm wide at anterior end, with rami ca. 60 μm long. Probable gonocoxites VIII (gonacoxapodemes?) visible as moderately sclerotized small oval lobes near posterior end of notum. Cerci short, hardly visible.

##### Paratype.

(Figs 1e–h, 2a). Visible characters as in holotype, with following additions. *Head* preserved only in PIN 4270/2367, 650 μm wide, 850 μm high with proboscis, ca. 400 μm high to lower eye margin. Eyes with medially narrowing dorsomedial extension, well separated by 60 μm. Coronal triangle ca. 130 μm high, ca. 100 μm wide; coronal suture clear, probably pair of poorly visible ocelli (ca. 30 μm in diameter) adjoining lower part ventrally to eye dorsomedial extension. Scape ca. 100 μm, pedicel ca. 40 μm in diameter. Flagellomeres (five distinctly visible lateral to eye margin) short-oval, 50–70 μm long, 30–40 μm wide. Clypeus ca. 200 μm wide, ca. 300 μm high. Proboscis ca. 550 μm long, sclerotized, stylet-like, pointed, with blade-like tapered laciniae and mandibles, labrum apically more sclerotized. Palpi poorly visible, looking widened (ca. 70 μm in distal parts); visible parts of palpi reaching about 4/5 of proboscis. *Thorax*. Scutum weakly, evenly convex; anterior part ca. 300 μm wide. Antepronotals narrowed medially. Scutellum ca. 120 μm long; postnotum ca. 200 μm long, 300 μm wide, with distinct longitudinal median groove. *Legs*. Measurements (μm). p1(?): ti 880, ta1-5 1200; p2: ti 1375, ta1 750–850, ta2 ca. 400, ta3-5 ca. 700, LR2 0.55; p3: fe 1000, ti 1450–1500, ta1-5 > 1250. Tibiae 60–100 μm wide near apex. Apices of mid-, hind tibiae with combs of separate spiniform setae (visible only in PIN 4270/2459): midtibia with no less than 3–4 slenderer setae ca. 50 μm long, hind tibia with no less than 3 thicker setae, longest ca. 50 μm. Hind tibia probably with two spurs, 70 and 50 μm long.

##### Remarks.

The pattern of the female mouthparts is poorly visible in the holotype and unknown in paratype PIN 4270/2459; paratype PIN 4270/2367 has a well-preserved proboscis and its wing venation is very pale and incomplete; the setae at the tibial apices are distinctly visible only in paratype PIN 4270/2459; in both paratypes the spermathecae are not visible. Hence it is possible that these specimens are not conspecific. However, we suggest that all these specimens belong to the same species due to visible peculiarities of venation (particularly the long Rs stem and R2 position).

The genus *Cretaenne* was described in the subfamily Aenneinae with reservations, due to the vein Sc not terminating in the wing margin and short Rs stem, as distinct from the type genus of the subfamily, *Aenne* Ansorge, 1999 from the Late Triassic and Early Jurassic of Europe (Krzeminski and Jarzembowski 1999, Azar et al. 2008). In the new species from Shar Teg, the stem Rs is long without doubt but Sc is clear only to the level of the first Rs bifurcation, then sharply thins out and possibly does not terminate in C, as seen in *Cretaenne*. We did not reexamine the type material of *Aenne* and *Cretaenne*, but according to the published data, Rs length can vary within species: in *Aenne liasina* Ansorge, 1999, the relatively long Rs is longer or shorter than *r-m* (Ansorge 1999: figs 6–7), whereas the distal thinning of Sc is not recorded. Consequently, the new species is described here as a member of *Cretaenne* due to several features unknown for *Aenne* (described from isolated wings only), viz. a reduced hind tibial comb, the postnotum with a longitudinal groove, the structure of well-developed extended proboscis, and, probably, spurs on middle and hind tibia. Actually, biting mandibles were reported for the Mesozoic *Aenne* with reference to unpublished data of Cranston (Grimaldi and Engel 2005: 504).

### Subfamily Podonominae Thienemann & Edwards, 1937

#### Genus Podonomius

Kalugina, 1985

http://species-id.net/wiki/Podonomius

Podonomius Kalugina and Kovalev 1985: 101. Type species *Podonomius tugnuicus*Kalugina and Kovalev 1985: 102.

##### Notes.

The genus was described for six species fromthe Early and Middle Jurassic of Siberia, with only two species being attributed with certainty. Later, one more species from the Early Jurassic of Germany was tentatively included (Ansorge 1996). The specimens under description are assigned to this genus due to their broadhead, large reniform eyes with dorsomedial projection, short thorax (no longer than its height), narrow scutellum, short postnotum, three rounded sclerotized spermathecae, and peculiarities of venation (vein C long, reaching R4+5 tip; Sc not terminating in wing margin; R2+3 absent; R1 long, not thickened distally in female; R4+5 straight or only slightly curved down distally; bR4+5 and *m-cu* inclined to long wing axis; *r-m*, bM3+4 and *m-c*u aligned; *r-m* much longer than bM3+4; cell *ba* and *bp* not symmetrical; *m-cu* connecting Cu proximal to *r-m*; costal and radial veins, stem M and CuA, *r-m*, bM3+4 and *m-cu* thickened, other veins very thin and pale; wings without spots).

We re-examined the type material of the six species described by Kalugina (1985) from Siberia (Fig. 2i) but did not examine ?*Podonomius tumidus* Ansorge, 1996 from Grimmen. A postnotum without longitudinal median groove was recorded in the original diagnosis of *Podonomius* (for German species, such information was absent; Ansorge 1996). This feature is known only in Aphroteniinae and Podonominae and unknown in Tanypodinae and Buchonomyiinae (Brundin 1966, Murray and Fittkau 1989, Sæther 1989). Unfortunately it is impossible to see this important character in the new specimens, in particular, due to the lateral position of impressions. Pubescence and setae of their bodies are also not visible on any specimens described here.

#### Podonomius blepharis sp. n.

urn:lsid:zoobank.org:act:EE993F1F-6B84-4BA7-80B6-9905C90DA6B2

http://species-id.net/wiki/Podonomius_blepharis

##### Etymology.

From Greek “blepharis” for eyelash, after the pattern of the tibial comb.

##### Material examined.

**Holotype**: part and counterpart of well-preserved female PIN 4270/2357±, SW Mongolia, Shar Teg (443/1); Late Jurassic.

##### Diagnosis.

The new species is distinguished by its small size (wing length l.4 mm), well-developed elongate proboscis, weakly convex scutum without a hump, wing with broad cell *c*, and pale legs with darker junction of femur with trochanter and tibia, and with combs of dark closely-spaced spiniform setae at tibial apices.

##### Description.

Female (Figs 1i–l, 2d–f). *Measurements* (mm): Total length 2.0; thorax length 0.8, height 0.9; abdomen length 1.0; wing length 1.4. Total length / wing length 1.4. *Coloration*. Head and thorax dark, abdomen lighter, legs pale with darker junction of femur with trochanter and tibia, with darker apices of tibiae. *Head* no less than 600 μm wide, no less than 550 μm high with proboscis, 380 μm high to lower eye margin. Eyes large, with wide dorsomedial extension, looking narrowly separated by ca. 30 μm. Facets equal. Coronal triangle ca. 100 μm high, coronal suture clear near upper eye margin. Scape 90 μm, pedicel 45 μm in diameter; proximal flagellomeres short-oval to rounded, ca. 35 μm wide and 40–50 μm long. Clypeus ca. 100 μm wide, ca. 150 μm high, possibly with longitudinal groove. Proboscis well-developed, elongate, sclerotized at least in distal part, ca. 200 μm long (projecting distally of clypeus for no less than 120 μm), ca. 30 μm wide at visible apex. *Thorax*. Scutum weakly, evenly convex, without hump or tubercle. Scutellum ca. 150 μm long, not projecting. Postnotum ca. 200 μm long. *Wing*. Vein C probably not produced beyond R4+5; cell *c* at *r-m* level subequal to cell *r1*, which only slightly narrower than *r5* cell at level of R1 tip; R1 approximately 2/3 as long as almost straight R4+5; *r-m* inclined to M. All veins mentioned strong, coloured. *Legs*. Measurements (μm). p1(?): ta1 380, ta2-5 ca. 405; p2: fe 700, ti 680; p3: fe 560, ti 680, ta1 ca. 500, ta2-5 ca. 650, LR3 ca. 0.75. Femora maximum ca. 110–120 μm wide, with thin sclerotized ridge ventrally near apex. Tibiae ca. 80 μm wide. Apices of mid-, hind tibiae with combs of dark closely-spaced spiniform setae ca. 50 μm long; in midtibial comb, no less than 10 setae, in hind tibial comb, 8 setae. Spurs not observed. *Abdomen*. Segments III–VII: tergites 150–180 μm long. Three large subequal oval moderately sclerotized spermathecae 100 μm long, ca. 80 μm wide, with necks (probably, long), in compact group. Sternite VIII with posteromedian sclerotized plate, its posterior margin bilobate; probable gonocoxites VIII (gonacoxapodemes?) visible as moderately sclerotized small oval lobes approximating each other. Cerci not visible.

##### Remarks.

The new species is similar to *Podonomius splendidus* Kalugina, 1985 (J1/2, Novospasskoye, Transbaikalia) in its venation (C length, ratio R1/ R4+5), colour pattern of legs and elongated mouthparts, which are visible on paratype PIN 3000/1857 (in the other type specimens of *Podonomius* from Siberia, the mouthparts are not visible due to the state of preservation). *Podonomius blepharis* sp. n. differs from *Podonomius splendidus* in broader cell *c* and smaller size. As for tibial combs, Kalugina noted (1985) that in *Podonomius tugnuicus* and *Podonomius splendidus* the tibial apices are darkened but without mentioning combs. According to our re-examination of the type material of *Podonomius splendidus*, the hind tibia has a reduced comb consisting of a row of separate dark points, which may be minute setae or possibly bases of missing long bristles (these seem to be visible near the tibial apex in the holotype). In the latter case, a well-developed tibial comb is not unique for *Podonomius blepharis*.

#### Podonomius macromastix sp. n.

urn:lsid:zoobank.org:act:AD0202E9-83D8-4462-B354-5D9697CA7485

http://species-id.net/wiki/Podonomius_macromastix

##### Etymology.

From Greek “makros” for long and “mastix” for whip, after the long antenna.

##### Material examined.

**Holotype**: part and counterpart of well-preserved female PIN 4270/2314±, SW Mongolia, Shar Teg (423/6); Late Jurassic.

##### Diagnosis.

The new species is distinguished by its small size (wing length 1.9), well-developed elongate proboscis, strongly convex scutum with a hump, comparatively long wings with R1 with arched tip and broad cell r5, and pale legs with darker junction of femur with trochanter and tibia.

##### Description.

Female (Figs 1m–n, 2g–h). *Measurements* (mm): Total length 2.0; thorax length 0.7, height 0.9; wing length 1.9, width 0.8; abdomen length 1.1. Total length / wing length ca. 1.05. *Coloration* pattern as in *Podonomius blepharis* sp. n. *Head* 480 μm wide, no less than 600 μm high with proboscis, 380 μm high to lower eye margin. Eyes large, with wide dorsomedial extension, narrowly separated by ca. 30 μm. Facets slightly increasing to lower eye parts. Coronal triangle ca. 50 μm high, coronal suture ca. 80 μm high. Antenna no less than 600 μm. Scape ca. 80 μm, pedicel ca. 60 μm in diameter; at least 12 flagellomeres, ca. 35 μm wide (proximal 6 flagellomeres moniliform, 40–45 μm long; others cylindrical, 50–70 μm long). Clypeus ca. 130 μm wide, ca. 150 μm high. Proboscis well-developed, elongated, tapering, ca. 180 μm long (about 1/3 of head height), ca. 70 μm wide at visible apex, with pair of separate sclerotized blades. *Thorax*. Scutum strongly convex, with hump before midlength, 700 μm long. Postnotum ca. 200 μm long. *Wing* much longer than abdomen. Vein C only slightly produced beyond R4+5, not reaching wing tip; cell *c* at *r-m* level broader than cell *r1*, which is almost half as wide as cell *r5* at R1 tip level; R1 with arched tip, 2/3 as long as slightly curved down distally R4+5; *r-m* slightly inclined, bM3+4 almost perpendicular to M. *Legs* poorly visible. Measurements (μm). p2(?): fe ca. 550, ti ca. 700, ta1-5 ca. 700; p3(?): ti ca. 870, ta1-5 > 800. Hind femora ca. 110 μm wide; hind tibiae 70–80 μm wide at apex, with sclerotized apical traces, possibly of setae bases. *Abdomen* with three large unequal short-oval sclerotized spermathecae 60–90 μm long, 50–60 μm wide, with necks, in compact group. Cerci ca. 40 μm long.

##### Remarks.

The new species is similar to ?*Podonomius rotundatus* Kalugina, 1985 (J2, Kubekovo, South Siberia, Fig. 2i) in its venation (length of C, R1/R4+5 ratio, cells r1/r5 ratio) and size, but is distinguished by the arched tip of R1. The new species differs from *Podonomius blepharis* sp. n. in the longer wings with broader cell r5 and thoracic shape.

#### ? Podonomius robustus sp. n.

urn:lsid:zoobank.org:act:189103E1-9FA9-45DE-A4E4-52EF0CE68913

http://species-id.net/wiki/Podonomius_robustus

##### Etymology.

From Latin “robustus” for stout, after the total habitus.

##### Material examined.

**Holotype**: Part and counterpart of partly preserved female PIN 4270/2254±, SW Mongolia, Shar Teg (443/1); Late Jurassic.

##### Diagnosis.

The new species is distinguished by its medium size (wing length 3.7 mm), well-developed, strongly elongate proboscis, wing with C produced beyond R4+5 and reaching wing tip, strongly sclerotized abdomen and legs, and one spermatheca situated proximally of other two.

##### Description.

**Figure 3.**
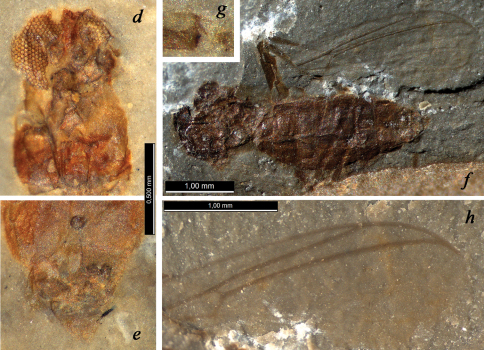
?*Podonomius robustus* sp. n., holotype (**a, d** head, positive impression **b, e** abdominal apex, negative impression **c, h** wing, negative impression **f** female habitus, negative impression **g** apex of hind tibia, positive impression; all photos made under alcohol except for **f**); Shar Teg, J3. Scale bar 0.5 mm

Female (Fig. 3). *Measurements* (mm): Total length 4.1; thorax length 1.5, width ca. 1.0; abdomen length 2.9; wing length 3.7. Total length / wing length 1.1. *Coloration*. Head, thorax, abdomen, legs uniformly dark. *Head* 700 μm wide, no less than 1100 μm high with proboscis, 550 μm high to lower eye margin. Eyes large, with wide dorsomedial extension, well-separated by ca. 100 μm. Facets equal. Frontal strip between eye extensions dark-coloured, long, sclerotized. Coronal triangle ca. 100 μm high, coronal suture ca. 70 μm high. Scape ca. 125 μm, pedicel ca. 40 μm in diameter. Clypeus ca. 200 μm high, ca. 150 μm wide. Proboscis very long, strong (longer than remainder of head), tapering, ca. 550 μm long, ca. 80 μm wide at (visible) apex; apical part with pair of sclerotized blades. Probable palpi no less than 400 μm long, two visible segments elongate, cylindrical, ca. 150 μm long, ca. 30 μm wide. *Thorax*. Scutum 900 μm long; postnotum ca. 250 μm long. *Wing* clearly longer than abdomen. Vein C produced beyond R4+5 (costal extension ca. 200 μm), reaching wing tip; cell *c* at *r-m* level broader than cell *r1*; cell *r5* at R1 tip level almost twice as wide as cell *r1*; R1 straight, 2/3 as long as R4+5. Vein C, radial veins, M, *r-m* strong and coloured; other veins thin, pale. *Legs*. Mid- and hind femora ca. 180 μm wide; tibiae 100–120 μm wide near apex. Apex of mid- or hind tibia with traces of at least 5 spiniform setae. *Abdomen*. Segments II–VIII 250–300 μm long, 800–900 μm wide. Three rounded sclerotized spermathecae 70–90 μm in diameter, with long necks, largest spermatheca situated proximally of other two. Gonapophysis IX distinctly visible, sclerotized, with notum ca. 200 μm long and rami ca. 50 μm long. Posterior margin of sternite VIII bilobate: probable gonocoxites VIII (gonacoxapodemes?) visible distal to spermathecae. Cerci distinct, elongate-oval, 150 μm long, ca. 70 μm wide.

##### Remarks.

Adults of this new species are the largest among *Podonomius* (wing length is similar only in ?*Podonomius simplex* Kalugina, 1985 (J2, Kubekovo, South Siberia), but in the Siberian species R1 is curved up distally). ?*Podonomius robustus* sp. n. differs from other species from Shar Teg also in the longer costal extension. Such a long costa extending to the wing tip is an important plesiomorphic character (Brundin 1976) and may be a character of generic value. Thus, the new species is only tentatively placed in *Podonomius*.

### Subfamily Tanypodinae Skuze, 1889

Among fifty adult chironomids from Shar Teg, only two incomplete females (PIN 4270/2324±, 4270/2431) and one male (PIN 4270/2384) can be determined as members of this subfamily (all from the same 443/1 outcrop) due to the typical venation on partly preserved wings (Fig. 1о). The poor state of their preservation does not allow us to place the specimens within a genus.

## Discussion

The four new species of Chironomidae described in this paper are characterized by the elongate proboscis with well-developed (probably sclerotized) mandibles and/or maxillae. To date, a well-developed piercing proboscis has been described only in two recent and no less than four extinct genera of the Chironomidae (Azar et al. 2008). Among the recent Chironomidae, strongly elongate mouthparts are known also in some Orthocladiinae, namely, in both sexes of the North American species *Pseudorthocladius macrostomus* Soponis, 1980 and *Rhinocladius* Edwards, 1931 (all three species; distributed in South America and Australia). Their proboscis, superficially resembling that of a mosquito, is formed entirely of the extremely elongated labellae, devoid of stylets and presumably used for sipping nectar, not for piercing (Edwards 1931, Freeman 1961, Soponis 1980). Possibly, a poorly described species *Camptocladius nigripectus* Bigot 1888 has a similar type of the proboscis (Edwards 1931).

The proboscises of *Cretaenne rasnicyni* sp. n. and ?*Podonomius robustus* sp. n. are much longer than in any other fossil Chironomidae described to date (possibly except for an undeterminable culicomorphan from the Triassic Cow Branch Formation; Blagoderov et al. 2007). Among the Chironomoidea, similar strongly elongate mouthparts are known in a number of recent species in many genera of Ceratopogonidae belonging to different lineages of this family, such as *Culicoides* Latreille, 1809, *Echinohelea* Macfie, 1940, *Atrichopogon* Kieffer, 1906, *Forcipomyia* Meigen, 1818, *Leptoconops* Skuse, 1889, as well as in extinct species such as the Lower Cretaceous *Protoculicoides skalskii* Szadziewski, Arillo, 1998, *Podonomius punctus* Borkent, 2000, and the Upper Cretaceous *Culicoides filipalpus* Remm, 1976 (e.g. Borkent 2000, Borkent et al. 2009). In nearly all Ceratopogonidae with such mouthparts, females are either insectivorous predators, or blood-suckers on vertebrates, or haemolymph-suckers on insects. However, haemolymph-sucking is restricted to Forcipomyiinae and considered derived feeding mode which appeared in the Cenozoic (Borkent 2000). Nectar-feeding ceratopogonids usually have the stylets more or less reduced, but *Forcipomyia (Forcipomyia) brevipennis* (Macquart, 1826) considered nectarophagous retains the sclerotized, distinctly toothed mandibles subequal in size to piercing mandibles of its insectivorous congeners (Glukhova 1981). By analogy with Ceratopogonidae we assume that the females of the new chironomid species were entomophagous or haematophagous but secondary nectarophagy cannot be excluded. It is impossible to argue for one of these feeding types, because the fine details of the mouthparts are not discernible in our fossils.

In the general appearance (very long and strong proboscis, body size, shape and proportions; pattern and degree of sclerotization, e.g. strongly sclerotized abdomen and legs) ?*Podonomius robustus* differs from “typical” Chironomidae as well as from other species assigned to the genus *Podonomius* andresembles some “robust” Ceratopogonidae, especially many Palpomyiini, but this advanced tribe is unknown from the Mesozoic (Szadziewski 1996). Unfortunately, the posterior part of the wing is not visible in the holotype of ?*Podonomius robustus*, as well as in the holotype of *Podonomius blepharis* sp. n., and the presence of a forked M1+2 (characteristic of Ceratopogonidae) cannot be excluded. Thus, the position of these two species seems to be somewhat uncertain. Similar female wings bare of macrotrichia, with well-developed single radial cell, costal ratio more than 0.9 and vein C produced beyond R4+5 and almost reaching wing tip, are known in several Cretaceous species of the ceratopogonid genus *Protoculicoides* Boesel, 1937, such as *Podonomius schleei* (Szadziewski, 1996) and *Podonomius unus* Borkent, 2000 from Lebanese amber (Borkent 2000).

However, in the holotype of ?*Podonomius robustus* the partly visible transverse vein under *r-m* is undoubtedly coloured, that is not recorded for the basal part of vein M2 in ceratopogonid wing, but typical for bM3+4 in podonomine wing (Figs 2h–i). In addition, the venation pattern of the anterior part of the wing is more similar to Podonominae than to Ceratopogonidae (vein R1 long, cell r1 long and not narrow) and the vertex of *Podonomius blepharis* as well as ?*Podonomius robustus* possesses a coronal suture, which is a feature of the Chironomidae, absent in Ceratopogonidae (Sæther 2000). Moreover, ?*Podonomius robustus* has a well-developed elongate notum of gonapophysis IX, whereas its absence has been considered as a synapomorphy of the Ceratopogonidae (Sæther 2000), and only some early lineages of Ceratopogonidae have a differently-shaped, short squat notum (vaginal apodeme in Borkent et al. 1987). So we exclude ceratopogonid affinity for both discussed species in spite of the incomplete state of preservation and the strong resemblance to insectivorous predatory or bloodsucking ceratopogonids in the general appearance and consider them as members of Podonominae.

The subfamily Podonominae is shown to be a dominant one in specimen abundance and diversity in the Jurassic deposits of Siberia (Kalugina and Kovalev 1985). No relevant data are available for the other regions. Kalugina stressed the difficulties in differentiation of the Mesozoic Podonominae and Tanypodinae and suggested that often it was possible to classify new taxa with certainty only as members of Tanypodoinae (Tanypodinae + Aphroteniinae + Podonominae) (Kalugina and Kovalev 1985: 82). However, she assigned new genera to subfamilies and explained her choice at every turn. At the same time, Kalugina assumed that some taxa she described in the Podonominae might actually belong to the Tanypodinae, considering that these two subfamilies were less clearly distinguished morphologically in the Jurassic.

Recently, Veltz et al. (2007) have concluded that subfamily identification of Jurassic Chironomidae is impossible and all Podonominae described by Kalugina are Chironomidae *incertae sedis*. Their only argument was rather methodical: Veltz and coauthors found it impossible to assign the different life stages to the same species as well as different species known only as pupae, to the same genus. Veltz and coauthors did not discuss the arguments proposed by Kalugina, e.g. that the larvae with translucent thoracic horns and pupae with translucent male genitalia were found among the numerous Jurassic impressions of *Oryctochlus* Kalugina, 1985, so the association of larvae with pupae was made with certainty and those of pupae and imago, with some doubts. Kalugina compared every life stage of *Oryctochlus* with those of recent *Trichotanypus* Kieffer, 1906 and drew a conclusion about an undoubted affinity of these two genera of Podonominae (e.g. in pupae of both genera, segment VIII is deeply emarginated posteriorly, which is remarkably similar to segment IX in shape and segment IX with 3 lateral setae, 2 of which are close together in mid-section). According to the time-calibrated molecular data (Cranston et al. 2010), *Trichotanypus* is one of the oldest genera of the subfamily, which split from Parochlini in the Early Cretaceous. The French authors did not discuss substantially any genus described by Kalugina. However, they considered that Podonominae may not be recorded in the Mesozoic (Azar et al. 2008), but that hardly can be true. A transfer of *Libanochlites* Brundin, 1976 (К1, Lebanese amber) from Podonominae to Tanypodinae made by these authors based on their new data was not supported by other specialists (Cranston et al. 2010).

## Supplementary Material

XML Treatment for Cretaenne

XML Treatment for Cretaenne rasnicyni

XML Treatment for Podonomius

XML Treatment for Podonomius blepharis

XML Treatment for Podonomius macromastix

XML Treatment for Podonomius robustus
